# Call to action: climate change and health threats to the Pacific Islands

**DOI:** 10.1186/s41182-025-00848-9

**Published:** 2026-01-03

**Authors:** Sara M. Damore, Caroline E. Ferguson Irlanda, Michele Barry

**Affiliations:** 1 Center for Human and Planetary Health, Stanford University, Stanford, CA 94305 USA; 2 Doerr School of Sustainability, Stanford University, Stanford, CA 94305 USA; 3 Center for Innovation in Global Health, Stanford University, Stanford, CA 94305 USA

**Keywords:** Pacific Island countries and territories, Climate change, Health impacts, Environmental determinants of health, Social determinants of health

## Abstract

The health impacts of climate change are increasingly evident in Pacific Island Countries and Territories (PICTs), a group of 22 nations facing significant and existential threats to their populations. This paper investigates the ways in which climate change exacerbates existing health issues in this vulnerable region, focusing on both communicable and non-communicable diseases, and the dynamic relationship between human and planetary health. Rapid urbanization, changes in food systems, and the ongoing epidemiological transition from infectious to chronic diseases reflect the complex interplay of colonization, globalization, and a changing climate. This paper reviews the unique climate challenges faced by PICTs, including rising sea levels, extreme weather events, and their impacts on food security, water resources, and healthcare. We explore the environmental and social determinants of health while highlighting how climate-induced changes compromise the health and well-being of communities throughout the Pacific region. We discuss the increasing prevalence of vector-borne and waterborne diseases, the exacerbation of the region’s immense noncommunicable disease burden, and the profound mental health impacts of climate change. The economic implications of these changes, particularly on tourism and fisheries, are also explored. Despite these challenges, PICTs have demonstrated remarkable resilience and remain at the forefront of global climate advocacy. This analysis underscores the urgent need for international solidarity and action to address climate change and protect the health and well-being of the vulnerable Pacific region.

## Introduction

The Pacific Island Countries and Territories (PICTs) are at the forefront of the impacts of climate change, despite contributing only 0.02% of global carbon emissions [1]. This disparity highlights the urgency in understanding and addressing the health implications of climate change in this vulnerable region. While conveying the risks of climate change to human health is often complicated by its varying impacts across different geographies, populations, and social strata, in the Pacific Islands these threats are decisive and existential.

The Pacific region encompasses 22 island nations spanning thousands of islands with a combined population of nearly 14 million (Table 1) [2]. Despite diverse cultures and geographies, these nations share similar epidemiological patterns and climate change vulnerabilities. This narrative review synthesizes current knowledge on climate change and health in PICTs, with particular attention to both direct health impacts and the complex indirect pathways via the social and environmental determinants of health that are often difficult to quantify.

**Table 1:** Demographics and health profiles of Pacific Island countries and territories

Country or Territory	Population (2023)	Land area (km^2^)	Political Status	Life expectancy, Females (2021)	Life expectancy, Males (2021)	Top 4 Causes of Death (2021)
American Samoa	47,521	222	Territory (USA)	72.8	72.4	Ischemic heart disease, diabetes, stroke, chronic kidney disease
Cook Islands	14,222	297	Free Association (New Zealand)	79.6	72.9	Diabetes, ischemic heart disease, stroke, hypertensive heart disease
Fed. States of Micronesia	112,630	799	Free Association (USA)	69.7	64.5	Ischemic heart disease, stroke, diabetes, chronic kidney disease
Fiji	924,145	20,857	Independent	68.8	63.8	Diabetes, ischemic heart disease, COVID-19, Stroke
French Polynesia	281,118	3939	Territory (France)	n.d.	n.d.	n.d.
Guam	166,506	588	Territory (USA)	82.9	73.4	Ischemic heart disease, COVID-19, stroke, lung cancer
Kiribati	132,530	995	Independent	67	61.1	Ischemic heart disease, diabetes, stroke, tuberculosis
Nauru	11,875	23	Independent	65.7	59.2	Ischemic heart disease, stroke, diabetes, lower respiratory infection
New Caledonia	289,870	21,613	Territory (France)	n.d.	n.d.	n.d.
Niue	1817	298	Free Association (New Zealand)	69.2	65.1	Ischemic heart disease, stroke, diabetes
Northern Mariana Islands	45,143	537	Territory (USA)	75	69.5	Ischemic heart disease, stroke, COVID-19, diabetes
Marshall Islands	38,827	286	Free Association (USA)	66.8	63.4	Ischemic heart disease, stroke, diabetes, tuberculosis
Palau	17,727	495	Free Association (USA)	70.5	67.7	Ischemic heart disease, stroke, diabetes, COPD
Papua New Guinea	10,389,635	67,754	Independent	65.5	61.9	COVID-19, stroke, ischemic heart disease, lower respiratory infection
Pitcairn Islands	57	47	Territory (United Kingdom)	n.d.	n.d.	n.d.
Samoa	216,663	3046	Independent	71.9	69.6	Ischemic heart disease, stroke, diabetes, COPD
Solomon Islands	800,005	29,675	Independent	68.4	63.7	Ischemic heart disease, stroke, lower respiratory infection, diabetes
Tokelau	2560 (2025)	16	Territory (New Zealand)	67.8	67.1	Ischemic heart disease, stroke, diabetes, lower respiratory infection
Tonga	104,597	847	Independent	75.7	70.6	Ischemic heart disease, diabetes, stroke, lower respiratory infection
Tuvalu	9816	44	Independent	70.6	65.8	Ischemic heart disease, stroke, diabetes, lower respiratory infection
Vanuatu	320,409	13,526	Independent	69.4	62.5	Ischemic heart disease, stroke, COVID-19, diabetes
Wallis and futuna	11,235	190	Territory (France)	n.d.	n.d.	n.d.

World Bank World Development Indicators, WHO Data, The Pacific Community, WHO Global Health Observatory, Institute for Health Metrics and Evaluation Country Profiles, and Nunn et al. (2020) [3–7]

*n.d.* no data available

Climate change is fundamentally altering weather patterns in the Pacific Islands through increased ocean warming and air temperatures, which alter oceanographic and atmospheric circulation patterns. These changes manifest by amplifying existing climate conditions through increased storm intensity, altered precipitation patterns, and rising sea levels [8]. The frequency and intensity of extreme weather events in PICTs have increased, consistent with climate change projections. This includes changes in rainfall patterns with exacerbated drought and flooding, higher air temperatures, and more intense tropical storms [9]. Cyclone Winston, which hit Fiji in 2016, was the strongest tropical cyclone on record to make landfall in the country, causing widespread destruction [10]. High tide flooding has also become more common; as a whole, the Pacific Islands have seen a median of 7 additional high tide flood days per year since 2020 [11].

Climate change implicates many health determinants, including air and water quality, food security, healthcare access, and economic stability. PICTs have formed a united front to advocate for a reduction in greenhouse gas emissions and take immediate action to mitigate the impacts of climate change [12]. PICTs demonstrate resilience and courage in the face of profound threats to their nations, emphasizing the need for international solidarity and action to address climate change’s disproportionate impact on this region.

This paper examines the magnitude of the direct and indirect health impacts of climate change in PICTs. It explores the complex epidemiological consequences of colonization and globalization while addressing how rising sea levels, extreme weather events, and disrupted weather patterns exacerbate and create novel challenges to health and well-being.

## Epidemiology

Health in PICTs has been significantly impacted by European colonization, World War II (including nuclear testing in French Polynesia and Marshall Islands), and increased globalization [13]. The epidemiological transition in this region from communicable to non-communicable diseases (NCDs) reflects these historical changes and their impact on lifestyle, diet, and economic development.

This transition is exemplified by the Solomon Islands, where cardiovascular disease has shifted from nearly non-existent to the country’s highest disease burden in 50 years, a pattern emblematic of the disease landscape across the region (Table 1) [14]. Diabetes is also a critical health challenge; in 2024, diabetes caused over one-third of deaths in Guam, New Caledonia, and French Polynesia—the highest burdens in the world [15]. This NCD epidemic is closely linked to changes in diet and physical activity as traditional lifestyles have given way to urbanized, sedentary behaviors and increased consumption of processed foods [13, 16].

While the globalization and commercialization of food systems in PICTs have prompted dramatic shifts in diets, many nations still struggle with the triple burden of undernutrition, overnutrition, and micronutrient deficiencies [17]. Stunting remains a significant issue, particularly in Melanesian countries such as Papua New Guinea, where the prevalence of stunting is 49.5% among children under five [18]. Simultaneously, rates of obesity and overweight have more than doubled in nearly every PICT over the past 50 years, further exacerbating the NCD burden [19].

Communicable diseases still present challenges in many nations; Tuberculosis (TB) remains a persistent threat; Marshall Islands, Kiribati, and Papua New Guinea all report some of the highest TB incidence rates in the world (692, 533, and 432 cases per 100,000, respectively) [20–22]. Other significant communicable diseases include leprosy (Kiribati), leptospirosis, HIV, STIs, typhoid fever, dengue fever, malaria, and Zika [23–25]. Efforts to prevent the re-emergence of recently eliminated diseases such as polio and measures are ongoing, as evidenced by the 2025 polio outbreak in Papua New Guinea, where the vaccination rate is around 44% [26].

These epidemiological shifts underscore the vulnerability of PICTs to rapid changes in physical and social environments, including those induced by climate change. Climate change exacerbates communicable and non-communicable disease incidence in PICTs through direct and indirect pathways implicating the social, physical, and environmental determinants of health, to be explored in greater depth in the later parts of this work. Moreover, the difficulty of data collection and management in these geographically dispersed and often resource-limited settings further complicates accurate assessment and response to disease risks and burdens, which are likely higher than reported due to under-reporting and incomplete case detection.

Pacific Island countries have systematically assessed and prioritized their climate-health vulnerabilities through national consultations (Table 2). While all countries recognize water security, food security, and vector-borne diseases as high-priority climate-sensitive health risks, there is notable variation in other identified priorities (note that the identified priorities from this 2015 assessment do not necessarily reflect current epidemiological or climate burdens). Mental health impacts and NCDs related to climate change, for example, are recognized by roughly half of surveyed nations. This variation reflects both differing exposures and potential gaps in awareness of emerging climate-health threats. The following sections examine these priority areas in depth.

**Table 2:** Highest-priority climate-sensitive risks identified by Pacific Island countries (2015)

Health risk category	COK	FJI	KIR	RMI	FSM	NRU	NIU	PLW	WSM	SLB	TON	TUV	VUT
Direct effects													
Extreme weather^a^	•	•	•	•	•	•	•	•	•	•	•	•	•
Heat-related illness^b^		•	•		•	•						•	
Indirect effects													
Water security^c^	•	•	•	•	•	•	•	•	•	•	•	•	•
Food security^d^	•	•	•	•	•	•	•	•	•	•	•	•	•
Vector-borne diseases^e^	•	•	•	•	•	•	•	•	•	•	•	•	•
Zoonoses^f^				•					•	•			
Respiratory illness^g^			•	•		•		•	•	•	•	•	•
Eye/ear//skin disorders^h^				•	•			•	•		•		•
Diffuse effects													
Mental health^i^		•			•			•	•	•			•
NCDs^j^		•			•	•		•	•	•			•
Health systems^k^		•	•	•	•	•				•		•	
Population pressure^l^												•	

Country codes: *COK* Cook Islands, *FJI* Fiji, *KIR* Kiribati, *RMI* (Republic of the) Marshall Islands, *FSM* Micronesia (Federated States of), *NRU* Nauru, *NIU* Niue, *PLW* Palau, *WSM* Samoa, *SLB* Solomon Islands, *TON* Tonga, *TUV* Tuvalu, *VUT* Vanuatu

^a^Health impacts of extreme weather events; includes traumatic injuries, deaths, and psychosocial impacts

^b^Including occupational exposure to hotter working conditions

^c^Water security and safety, including waterborne diseases causing diarrheal illness, typhoid fever, and sea-level rise-induced salination of water supplies

^d^Food security and safety, including malnutrition, foodborne diseases, and ciguatera fish poisoning

^e^Including dengue fever and malaria (currently limited to Solomon Islands and Vanuatu)

^f^Primary zoonosis of concern is leptospirosis

^g^Including respiratory infections, asthma, and pulmonary effects of heat and air pollution

^h^Range of problems from skin infections and cataracts to sexually transmitted infections

^i^Mental/psychosocial health disorders, including depression, anxiety, and PTSD from loss of life, land, or livelihoods

^j^Primarily cardiovascular disease, cerebrovascular disease, hypertension, and diabetes; may include cancers and mental health disorders

^k^Compromised access to health services, damage to health infrastructure, and strain on resources for disease surveillance

^l^Climate change-induced resettlement and sea-level rise exacerbating overcrowding

Source: Reproduced from WHO Regional Office for the Western Pacific (2021) based on data from WHO Regional Office for the Western Pacific (2015) [27, 28]

## Communicable diseases

As extreme weather, warming air temperatures, and flooding events expand mosquito breeding sites, arbovirus cases are rising throughout the region. From 1990 to 2019, dengue outbreaks doubled in Nauru, Kiribati, and the Solomon Islands and rose substantially in Vanuatu and Papua New Guinea, where disease prevalence has historically remained low [29–31]. Similarly, despite eradication attempts, malaria incidence has increased in several PICTs, including Solomon Islands, where estimated absolute cases among the population rose from approximately 40,000 to 180,000 between 2014 and 2023 [32, 33]. This pattern is mirrored in the transmission of Zika and Chikungunya, both of which were first documented in the Pacific within the last 20 years and have since expanded their ranges significantly due to changing climatic conditions and increased human mobility [33, 34, 35]. Lymphatic filariasis (elephantiasis) transmission also persists throughout the Region despite extensive eradication efforts, a trend that may be exacerbated by the expansion in range of its mosquito vectors [36, 37].

Waterborne diseases also present a significant threat, particularly following extreme weather events which can devastate sanitation facilities and limit freshwater resources. In Tuvalu and most other Pacific atolls, rainwater harvesting is the primary source of the country’s freshwater supply, increasing their susceptibility to drought-induced water scarcity and waterborne diseases [38, 39]. Drought frequency and intensity are increasing across the Pacific, with the Pacific Small Island Developing States (SIDS) experiencing the largest increases in extreme drought among SIDS subregions and projections indicating more extreme rainfall events and subsequent flooding [40, 41]. In Fiji, post-cyclone outbreaks are estimated to have caused 279 suspected cases of typhoid following cyclone Thomas and 1217 suspected cases of leptospirosis following flood events in 2012 [42]. A phylogenomic analysis of *Salmonella* Typhi in Fiji reports that post-cyclone typhoid cases were driven by event-related displacement [43].

Previous studies in the Pacific have noted increased rates of diarrheal episodes alongside increased annual temperature, extreme rainfall, and decreased freshwater availability, dynamics which are worsened by climate change [44]. In 2014, the Solomon Islands experienced a significant outbreak of diarrhea following a large flooding event, affecting over 6000 people [45]. A lack of water during drought can also increase diarrhea transmission, as evidenced in Tuvalu in 2011 [38]. These post-disaster disease outbreaks often overwhelm health systems already strained by storm damage and limited resources [46].

Climate change is also impacting the prevalence and distribution of *Vibrio* species, bacteria found in coastal waters and estuaries that can cause illness through the consumption of contaminated seafood or exposure to open wounds. Warming seas and extreme weather events are creating favorable conditions for the proliferation of *Vibrio* species, which are expected to continue to spread under current climate conditions [47].

## Noncommunicable diseases

Noncommunicable diseases—namely, cardiovascular disease, diabetes, cancer, and chronic respiratory diseases—are the leading cause of death in nearly every PICT [48]. Climate change affects NCDs through multiple pathways, including heat exposure, air quality degradation, climate-related mental health impacts, and disruptions to health systems and food security.

Cardiovascular diseases (CVDs) are the leading cause of mortality in the Pacific, where they are found at some of the highest rates in the world [48]. Temperatures are rising across PICTs, with hotter days and nights increasingly affecting human health. Between 2018 and 2022, people aged 65 and older in SIDS including PICTs were exposed to an average of 103 health-threatening heat days per year, nearly double the average of 53 days between 1998 and 2002 [40]. While there is variability in the exposure to extreme heat among PICTs, Guam is notably affected with the number of days exceeding 88°F increasing from 5 days per year in the 1950s to 36 days in the 1990s, and projections under high emissions scenarios predicting up to 257 days per year above 90°F by the end of this century [49]. Heat exposure is strongly associated with increased cardiovascular morbidity and mortality and the combined effects of heat and existing air pollution increase the likelihood of myocardial infarction [50]. Air quality is already a significant concern in the region due to waste burning, emissions from vehicles and generators (which further contribute to climate change), and cooking over open flames [51]. Moreover, shared risk factors and comorbidities for CVD and diabetes, including obesity, hypertension, and physical inactivity, are exacerbated by the impacts of climate change on agricultural productivity and fisheries, to be discussed in the following Sects. [52].

Climate change also exacerbates chronic respiratory diseases, which are in the top 5 causes of death in most PICTs [53]. Drought and flood intensity are increasing across the Pacific [41]. Flooding promotes mold growth, which can worsen chronic bronchitis, asthma, and Chronic Obstructive Pulmonary Disease (COPD), though more Pacific-specific data are needed to examine the intensity of these impacts in PICTs [54].

Sea level rise presents significant risk to coastal areas in PICTs, with devastating effects on low-lying nations [8]. While rising sea levels have varying effects on all Pacific Islands, atoll islands, such as Kiribati, Tuvalu, and Marshall Islands, where the average elevation is less than 3 m above sea level, are at risk of submersion from rising sea levels. In Solomon Islands, where five vegetated reef islands have already disappeared due to sea-level rise and erosion, 98% of participants reported daily concerns about sea level rise [55, 56]. In Tuvalu, 95 out of 100 interviewed participants reported significant distress from climate change [57]. These mental health impacts of climate change are compounded by existing high rates of suicide, limited mental health infrastructure, and a prevailing mental health stigma in many Pacific communities [58]. Negative emotions and worsened mental health due to the loss of connection to land and ancestors, aspects that are fundamental to identity, culture, and spiritual well-being, are documented in Pacific people throughout the region [55, 57]. After cyclones Judy and Kevin in Vanuatu, a reported 19.6% of households were displaced and 7.5% affected by serious illness or injury [59]. Climate-induced migration and displacement create barriers to key social determinants of health; employment, housing, healthcare, and sociocultural establishment [55]. Mental health impacts may also manifest in violence and injuries, with increasing evidence linking warmer temperatures and extreme weather to violence and conflict [60–62]. Given the unique vulnerabilities of PICTs to climate change and mental health disruptions, there is an urgent need for comprehensive research and mental health capacity building in the region.

Extreme weather events damage health infrastructure, disrupt medication supply chains, and prevent patients from accessing care [63]. Economic losses strain household resources, reducing ability to pay for health services. Given the complexity and high prevalence of NCDs in PICTs amid the region’s unique vulnerability to climate change, there is an urgent need for climate-resilient health systems that consider chronic care.

The impacts of climate change on NCDs are mediated by social and environmental determinants of health. However, critical data gaps in heat exposure, air quality monitoring, and climate-health linkages across most PICTs limit comprehensive understanding of the full scope of these direct and indirect impacts. Addressing NCDs—the most significant epidemiological burden in PICTs—in the context of climate change will require improved surveillance, more robust data collection, and cross-sectoral approaches that strengthen health systems, improve chronic disease prevention and management, and build community resilience to climate impacts.

## Food systems

Traditional diets in the Pacific comprise plant foods, such as taro, tapioca, breadfruit, coconut, sweet potato, and banana, as well as fish and marine invertebrates. This diet has been a source of nutritional security and cultural identity for millennia and has endured centuries of colonization [64]. Food sharing practices are the foundation of the social fabric of Pacific communities, with reciprocal exchange networks yielding food system resilience during times of crisis [65].

In recent decades, PICTs have undergone a nutrition transition, consuming fewer traditional foods and more imported foods, altering their food and nutritional security landscape [66]. Colonization, the introduction of new religions, and the spread of capitalism have led to declines in crop diversity, food storage and preservation, famine foods, and the strength of networks [64]. The scale of this dependence varies across islands; in one extreme, approximately 80% of dietary intake in Kiribati comes from imported foods [67]. This shift has profound health implications, directly contributing to the rising rates of NCDs and their risk factors, such as diabetes, cardiovascular disease, hypertension, and obesity [68]. The economic and nutritional vulnerability created by this import dependence exposes communities to supply chain disruptions and price volatility, making them particularly susceptible to global market shocks [65]. Climate change further compounds these food security challenges through both immediate and long-term impacts. Extreme weather events cause acute food shortages and damage to boats and gear that can persist for months or years after the initial impact [69]. These events have significant nutritional implications, reducing access to local nutritious foods while increasing reliance on imported nutrient-poor alternatives, reinforcing the cycle of diet-related NCDs [16].

The Pacific marine environment faces a triple burden of environmental constraints: sea level rise, ocean warming, and ocean acidification are intensifying, threatening an ecological stability that is central to life and food systems in PICTs. Sea surface temperatures in the Pacific have risen by approximately 0.7 °C since the 1950s. In the Southwest Pacific, this warming is happening nearly three times faster than in the global average [1]. Marine foods represent 50–90% of dietary animal protein in the region; changes to local fish populations resulting from climate-driven oceanographic shifts impact food security in the islands and could exacerbate the reliance on imported, heavily processed foods which contribute to the NCD epidemic in the Region [70]. Ocean warming has triggered widespread coral bleaching events, including a loss of up to 80% of the coral cover in Kiribati during the 2015–2016 global coral bleaching event [71]. Bleaching events can kill coral, which play an integral role in the marine food chain and physical marine habitat, creating cascading ecosystem effects throughout marine food webs [72]. The productivity of reef fisheries in some PICTs is projected to decline by as much as 50% by the 2050s under continued high greenhouse gas emissions [73]. Ocean acidification poses additional threats to shellfish and coral-dependent species [74]. Climate change also has the potential to cause range shifts in fish populations, affecting local availability of food fish [75]. In addition, warming oceans are associated with increased incidence of Ciguatera poisoning, a foodborne illness caused by eating contaminated reef fish, presenting an additional threat to island food systems reliant on local reef fish [76, 77].

As for terrestrial foods, around 80% of all Pacific Islanders rely on agricultural produce from their own gardens or from smallholder farmers to support or to supplement their diets [78]. Saltwater intrusion represents an immediate threat, contaminating both freshwater sources and agricultural soils essential for crop cultivation and potentially leading to hypertension [79]. Erosion and sea level rise threaten the permanent loss of arable land, particularly in low-lying atolls [80]. Changing precipitation patterns are creating new challenges, with some areas experiencing severe droughts, while others face destructive flooding, both of which can significantly impact crop yields and food security [81].

Pacific communities have demonstrated remarkable adaptive capacity across generations. Food sharing networks continue to support food security and sovereignty, and help communities to redistribute resources during times of scarcity and maintain social cohesion [65, 82, 83]. Communities show flexibility in shifting between fishing and agriculture based on changing environmental conditions and resource availability [65].

## Impacts on health systems

The increased frequency and intensity of natural disasters strain health infrastructure and community resilience. A spatial analysis in 14 PICTs found that 62% of health facilities were located within 500 m of the coast, highlighting their vulnerability to sea-level rise and storm surges [84]. This coastal proximity, combined with existing barriers to accessing health services, poses significant challenges for healthcare access and emergency response during extreme weather events. In Solomon Islands, for instance, 39% of rural households take more than 1 h to reach a healthcare facility, with 67% of households nationally relying on walking as their primary mode of transport to health services, and 16% of rural households depending on canoes [85].

In addition, many PICTs rely on limited medical infrastructure and face a shortage of healthcare providers. In Tuvalu, the population of approximately 10,000 is served by 150-bed hospital [86]. These facilities are at particular risk of being overwhelmed by post-disaster communicable disease outbreaks, creating an immediate health threat while simultaneously disrupting continuing care for chronic disease management.

The current state of climate-preparedness in PICTs varies, but many health systems remain underprepared for the escalating impacts of climate change [87]. This challenge is further complicated by the region’s exceptional geographic isolation and diversity. International organizations and foreign aid are providing support for these efforts through funding and technical assistance. However, significant gaps remain in sustainable financing, technological capacity, and human resources [87]. Addressing these challenges is crucial to ensuring that PICTs can adequately serve their populations, especially amid increased risks of communicable disease outbreaks and the continuing need for chronic disease management.

## Climate-sensitive economic sectors and health

Climate change threatens the economic stability of Pacific Island communities by impacting climate-sensitive sectors, such as agriculture, fisheries and tourism. These industries influence population health through food systems, household income available for healthcare and nutrition, and government revenue that funds health infrastructure services. In Vanuatu, for example, approximately 65% of Gross Domestic Product (GDP) is linked to tourism and 20% to agriculture. In 2015, Cyclone Pam caused an estimated VT 48.6 billion (US$449.4 million) in damage and loss, equivalent to 64.1% of the country’s GDP [88]. In 2023, it was reported that 64.9% of surveyed households were affected by economic and livelihood losses due to the emergency following cyclones Judy and Kevin, 36.6% of which was due to lost harvest or ability to plant crops [59]. These economic shocks compromise household food security, nutrition, and access to health services.

Beyond extreme weather events, PICT economies are threatened by fisheries losses due to ocean warming and acidification. Many Pacific Island nations rely heavily on fishing license revenues as a source of revenue (in Kiribati, for example, licenses to foreign fishing vessels comprise 72% of the country’s GDP) [89]. Declines in fisheries productivity reduce national revenue available for health infrastructure and household access to affordable, nutritious protein sources. Coral reef degradation from ocean warming and acidification further threatens both fisheries and tourism sectors [90]. The combination of economic instability in climate-sensitive industries and fragile health systems creates a reinforcing cycle in which climate impacts compromise both the financial resources needed to support health services and the nutritional foundations of population health.

## Climate action and advocacy

As the impacts of climate change have unfolded in PICTs, national and regional authorities have responded by developing climate and health adaptation strategies. The World Health Organization (WHO) Western Pacific Regional Office’s *Pacific Islands Action Plan on Climate Change and Health* (2018) provides a framework for building engagement with national and international actors, strengthening health system resilience, addressing climate-sensitive diseases, and improving access to sustainable climate and health financing [91]. This framework supports countries in mainstreaming climate change adaptation into national policies and plans. However, implementation of these plans faces challenges due to limited financial resources, technical capacity, and competing development priorities [92–94].

PICTs have been at the forefront of global climate advocacy, recognizing that mitigation is essential for protecting health, culture, and national sovereignty. The Marshallese delegation successfully advocated for the 1.5 °C warming target in the Paris Agreement, rallying around “1.5 to stay alive,” a target driven by the existential health threats facing low-lying island nations [95]. Fiji was the first country to ratify the agreement. In 2023, Vanuatu led 132 nations in requesting an International Court of Justice advisory opinion on States’ climate obligations, which could accelerate redress of climate-related losses and damages [96].

Despite these efforts, significant gaps remain between policy commitments and health protection on the ground. The region requires sustained international support—including climate finance, technology transfer, and capacity building—to translate adaptation plans into effective health outcomes.

## Remaining uncertainties

Despite these actions, significant uncertainties remain in understanding and addressing the full scope of the impacts of climate change in PICTs. Limited data collection, analysis, and availability hinder the ability to comprehensively understand the changing circumstances in the region and there is a critical need for more research informed by Pacific Islanders on the lived experience of climate change in the region (Table 1). Furthermore, the long-term mental health impacts of climate change and migration are not fully understood, especially within Pacific cultural contexts. Finally, the long-term effectiveness of current adaptation strategies in the face of accelerating climate change remains uncertain. As the world comes to terms with the full impacts of climate change, addressing these uncertainties is crucial to developing effective, culturally conducive strategies to protect the health and well-being of Pacific Island communities.

The health impacts of climate change in PICTs arise from complex, interlinked social and environmental systems. Accordingly, solutions require transdisciplinary, multisectoral collaboration, community-led design, resilient financing, and adaptive implementation. This work represents a necessary step toward more granular, country- and community-specific assessments that can guide tailored interventions and policy. Echoing other significant calls to action, immediate priorities include strengthening vector control, climate-resilient health systems, fortifying local food pathways, and, most importantly, a reduction in global emissions. Protecting the health and well-being of Pacific Island communities will require sustained commitment to integrated, multilevel action that addresses both the direct and indirect pathways through which climate change affects health.

## Data Availability

No datasets were generated or analysed during the current study.

## Abbreviations

COPD: Chronic obstructive pulmonary disease
CVD: Cardiovascular disease
GDP: Gross domestic product
NCD: Noncommunicable disease
PICTs: Pacific Island Countries and Territories
SIDS: Small Island Developing States
TB: Tuberculosis
WHO: World Health Organization

## References

[1] World Meteorological Organization. Climate change transforms Pacific Islands. Geneva: WMO. 2024. https://wmo.int/news/media-centre/climate-change-transforms-pacific-islands. Accessed 30 Jun 2025.

[2] Andrew NL, Bright P, Rua L, Teoh SJ, Vickers M. Coastal proximity of populations in 22 Pacific Island Countries and Territories. PLoS ONE. 2019;14(9):e0223249. 31568527 10.1371/journal.pone.0223249 PMC6768456

[3] World Bank. World development indicators. Washington, DC: World Bank; 2024. https://databank.worldbank.org/source/world-development-indicators. Accessed 30 Jun 2025.

[4] World Health Organization. WHO Data. Geneva: WHO; 2024. https://data.who.int/indicators. Accessed 2 Oct 2025.

[5] Institute for Health Metrics and Evaluation. Health research by location. Seattle: IHME; 2024. https://www.healthdata.org/research-analysis/health-by-location/profiles. Accessed 2 Oct 2025.

[6] World Health Organization. Global Health Observatory. Geneva: WHO; 2024. https://www.who.int/data/gho. Accessed 30 Jun 2025.

[7] Nunn PD, Kumar L, McLean R, Eliot I. Islands in the Pacific: settings, distribution and classification. In: Kumar L, editor. Climate change and impacts in the Pacific. Cham: Springer International Publishing; 2020. p. 33–170.

[8] Mycoo M, Wairiu M, Campbell D, Duvat V, Golbuu Y, Maharaj S, et al. Small Islands. In: Pörtner H-O, Roberts DC, Tignor M, Poloczanska ES, Mintenbeck K, Alegría A, et al., editors. Climate change 2022: impacts, adaptation and vulnerability. Contribution of working group II to the sixth assessment report of the intergovernmental panel on climate change. Cambridge: Cambridge University Press; 2022. p. 2043–121.

[9] Dong C, Noyelle R, Messori G, Gualandi A, Fery L, Yiou P, et al. Indo-Pacific regional extremes aggravated by changes in tropical weather patterns. Nat Geosci. 2024;17(10):979–86.

[10] The Pacific Community. WACOP wave atlas: severe tropical cyclone Winston inundation. Noumea: SPC; 2024. https://wacop.gsd.spc.int/STC_Winston_inundation.html. Accessed 30 Jun 2025.

[11] NOAA. Annual high tide flooding outlook. Silver Spring, MD: National Oceanic and Atmospheric Administration; 2024. https://tidesandcurrents.noaa.gov/high-tide-flooding/annual-outlook.html. Accessed 30 Jun 2025.

[12] Pacific Islands Forum Secretariat. (2019). Kainaki II Declaration for Urgent Climate Change Action Now [PDF]. Retrieved from https://forumsec.org/sites/default/files/2024-03/2019%20Kainaki20II%20Declaration%20for%20Urgent%20Climate%20Change%20Action%20Now.pdf

[13] WHO Regional Office for the Western Pacific. Noncommunicable diseases and mental health in the Pacific: an urgent need for action. Manila: WHO Regional Office for the Western Pacific; 2022. https://iris.who.int/handle/10665/364534. Accessed 30 Jun 2025.

[14] Prior IA, Evans JG, Harvey HP, Davidson F, Lindsey M. Antecedents of cardiovascular disease in six Solomon Islands societies. Circulation. 1974;49(6):1132–46. 4831656 10.1161/01.cir.49.6.1132

[15] International Diabetes Federation. IDF diabetes atlas 2025. 11th ed. Brussels: IDF; 2025. https://diabetesatlas.org/resources/idf-diabetes-atlas-2025/. Accessed 30 Jun 2025.

[16] Frazier AG, Johnson MVV, Fortini LB, Giardina CP, Grecni ZN, Kane HH, et al. Hawai’i and US-affiliated Pacific Islands. In: Crimmins AR, Avery CW, Easterling DR, Kunkel KE, Stewart BC, Maycock TK, editors., et al., Fifth national climate assessment. Washington, DC: U.S. Global Change Research Program; 2023. p. 1638–68.

[17] Hughes S, Yau A, Max L, Petrovic N, Davenport F, Marshall M, et al. The triple burden of malnutrition among children in sub-Saharan Africa and South Asia: a comprehensive review. Nutr Rev. 2020;78(12):1207–17.

[18] Global Nutrition Report. Country Nutrition Profiles—Papua New Guinea. Bristol: Development Initiatives; 2024. https://globalnutritionreport.org/resources/nutrition-profiles/oceania/melanesia/papua-new-guinea/. Accessed 30 Jun 2025.

[19] World Health Organization. Global Health Observatory data repository. Geneva: WHO; 2024. http://www.who.int/gho/en/. Accessed 30 Jun 2025.

[20] World Health Organization. WHO data country profile: Marshall Islands. Geneva: WHO; 2024. https://data.who.int/countries/584. Accessed 30 Jun 2025.

[21] World Health Organization. WHO Data Country Profile: Kiribati. Geneva: WHO; 2024. https://data.who.int/countries/296. Accessed 30 Jun 2025.

[22] World Health Organization. WHO data country profile: Papua New Guinea. Geneva: WHO; 2024. https://data.who.int/countries/598. Accessed 30 Jun 2025.

[23] Costa F, Hagan JE, Calcagno J, Kane M, Torgerson P, Martinez-Silveira MS, et al. Global morbidity and mortality of leptospirosis: a systematic review. PLoS Negl Trop Dis. 2015;9(9):e0003898. 26379143 10.1371/journal.pntd.0003898 PMC4574773

[24] World Health Organization. Global leprosy (Hansen disease) update, 2020: impact of COVID-19 on global leprosy control. Wkly Epidemiol Rec. 2021;96(36):421–44.

[25] World Health Organization Western Pacific Region. Combatting communicable diseases in the Pacific. Manila: WHO WPRO; 2024. https://www.who.int/westernpacific/activities/combatting-communicable-diseases-in-the-pacific. Accessed 30 Jun 2025.

[26] World Health Organization. Circulating vaccine-derived poliovirus type 2 (cVDPV2)—Papua New Guinea. Geneva: WHO; 2025. https://www.who.int/emergencies/disease-outbreak-news/item/2025-DON571. Accessed 30 Jun 2025.

[27] World Health Organization Regional Office for the Western Pacific. Climate change and health in Small Island Developing States: a WHO special initiative, Pacific island countries and areas. Manila: WHO Regional Office for the Western Pacific; 2018. https://iris.who.int/server/api/core/bitstreams/e8ed112e-5a7e-4fe0-8d1c-e4f18160a2fb/content.

[28] World Health Organization Regional Office for the Western Pacific. Human health and climate change in Pacific island countries. Manila: WHO Regional Office for the Western Pacific; 2015. http://iris.wpro.who.int/bitstream/handle/10665.1/12399/9789290617303_eng.pdf.

[29] Nakase T, Giovanetti M, Obolski U, Lourenço J. Population at risk of dengue virus transmission has increased due to coupled climate factors and population growth. Commun Earth Environ. 2024;5(1):475.

[30] Filho WL, Scheday S, Boenecke J, Gogoi A, Maharaj A, Korovou S. Climate change, health and mosquito-borne diseases: trends and implications to the Pacific region. Int J Environ Res Public Health. 2019;16(24):5114. 31847373 10.3390/ijerph16245114 PMC6950258

[31] Feng F, Ma Y, Qin P, Zhao Y, Liu Z, Wang W, et al. Temperature-driven dengue transmission in a changing climate: patterns, trends, and future projections. Geohealth. 2024;8(10):e2024GH001059. 10.1029/2024GH001059 PMC11436633 39347019

[32] Sorenson J, Watkins AB, Kuleshov Y. The influence of climate variables on malaria incidence in Vanuatu. Clim. 2025;13(2):22.

[33] World Health Organization. World Malaria Report 2024: Solomon Islands Country Profile. Geneva: WHO; 2024. https://cdn.who.int/media/docs/default-source/country-profiles/malaria/malaria-2024-slb.pdf. . Accessed 30 Jun 2025.

[34] Duffy MR, Chen TH, Hancock WT, Powers AM, Kool JL, Lanciotti RS, et al. Zika virus outbreak on Yap Island, Federated States of Micronesia. N Engl J Med. 2009;360(24):2536–43. 19516034 10.1056/NEJMoa0805715

[35] Silva LA, Dermody TS. Chikungunya virus: epidemiology, replication, disease mechanisms, and prospective intervention strategies. J Clin Invest. 2017;127(3):737–49. 28248203 10.1172/JCI84417 PMC5330729

[36] Yajima A, Ichimori K. Progress in the elimination of lymphatic filariasis in the Western Pacific Region: successes and challenges. Int Health. 2020;13(Suppl 1):S10–6. 33349886 10.1093/inthealth/ihaa087 PMC7753160

[37] Lafferty KD. The ecology of climate change and infectious diseases. Ecology. 2009;90(4):888–900. 19449681 10.1890/08-0079.1

[38] Emont JP, Ko AI, Homasi-Paelate A, Ituaso-Conway N, Nilles EJ. Epidemiological investigation of a diarrhea outbreak in the South Pacific island nation of Tuvalu during a severe La Niña-associated drought emergency in 2011. Am J Trop Med Hyg. 2017;96(3):576–82. 28138046 10.4269/ajtmh.16-0812 PMC5361530

[39] UNICEF Pacific Islands. Consolidated Emergency Report 2022: Pacific Islands. Suva: UNICEF; 2023. https://open.unicef.org/sites/transparency/files/2023-05/Pacific%20Islands%20CER%202022.pdf.

[40] The Lancet Regional Health—Western Pacific. The 2024 report of the Lancet Countdown on health and climate change for Small Island Developing States: falling through the cracks. Lancet Reg Health West Pac. 2024;52:101209.39430124

[41] Connell J. Impacts of climate change on settlements and infrastructure relevant to the Pacific Islands. In: Seneviratne L, editor. Pacific marine climate change report card: science review 2018. Apia: SPREP; 2018. p. 159–76.

[42] Ritter J, Lau C, Craig S, Goarant C. A large leptospirosis outbreak following successive severe floods in Fiji, 2012. Am J Trop Med Hyg. 2018;99(4):1165–8. 30141390 10.4269/ajtmh.18-0335 PMC6159581

[43] Davies MR, Duchene S, Valcanis M, Jenkins AP, Jenney A, Rosa V, et al. Genomic epidemiology of Salmonella Typhi in Central Division, Fiji, 2012 to 2016. Lancet Reg Health West Pac. 2022;24:100444. 10.1016/j.lanwpc.2022.100488 PMC9234096 35769175

[44] Singh RB, Hales S, de Wet N, Raj R, Hearnden M, Weinstein P. The influence of climate variation and change on diarrheal disease in the Pacific Islands. Environ Health Perspect. 2001;109(2):155–9. 11266326 10.1289/ehp.01109155 PMC1240636

[45] Jones FK, Ko AI, Becha C, Joshua C, Musto J, Thomas S, et al. Increased rotavirus prevalence in diarrheal outbreak precipitated by localized flooding, Solomon Islands, 2014. Emerg Infect Dis. 2016;22(5):875–9. 27088272 10.3201/eid2205.151743 PMC4861519

[46] World Health Organization Western Pacific Region. A month after Cyclone Pam, Vanuatu continues to face health challenges. Manila: WHO WPRO; 2015. https://www.who.int/westernpacific/about/how-we-work/pacific-support/news/detail/15-04-2015-a-month-after-cyclone-pam-vanuatu-continues-to-face-health-challenges. Accessed 2 Jan 2025.

[47] Baker-Austin C, Lake I, Archer E, Hartnell R, Trinanes J, Martinez-Urtaza J. Stemming the rising tide of *Vibrio* disease. Lancet Planet Health. 2024;8(7):e515–20. 38969478 10.1016/S2542-5196(24)00124-4

[48] World Bank. Pacific possible: health and noncommunicable diseases. Washington, DC: World Bank; 2017. https://thedocs.worldbank.org/en/doc/143531466064202659-0070022016/original/pacificpossiblehealthsummary.pdf. Accessed 30 Jun 2025.

[49] Keener VW, Grecni Z, Finucane M, Leong JA, Miles W, Wetzler R, editors. Climate Change in Guam: Indicators and Considerations for Key Sectors. Honolulu: East-West Center; 2020. https://www.eastwestcenter.org/publications/climate-change-in-guam-indicators-and-considerations-key-sectors.

[50] Xu R, Huang S, Shi C, Wang R, Liu T, Li Y, et al. Extreme temperature events, fine particulate matter, and myocardial infarction mortality. Circulation. 2023;148(4):312–23. 37486993 10.1161/CIRCULATIONAHA.122.063504

[51] Hilly JJ, Sinha J, Mani FS, Turagabeci A, Jagals P, Thomas DSG, et al. PM2.5 and PM10 concentrations in urban and peri-urban environments of two Pacific Island Countries. Atmos Pollut Res. 2025;16(5):102454.

[52] Andrew NL, Allison EH, Brewer T, Connell J, Eriksson H, Eurich JG, et al. Continuity and change in the contemporary Pacific food system. Glob Food Secur. 2022;32:100608.

[53] World Health Organization Regional Office for the Western Pacific. Regional action framework for noncommunicable disease prevention and control. Manila: WHO Regional Office for the Western Pacific; 2014. https://www.who.int/publications/i/item/9789290620044.

[54] Peirce AM, Espira LM, Larson PS. Climate change related catastrophic rainfall events and non-communicable respiratory disease: a systematic review of the literature. Climate. 2022;10(7):101. 10.3390/cli10070101.

[55] Mengesha NA, Sarnyai Z. The mental health impact of climate change on Pacific Islanders: a systematic review focused on sea level rise and extreme weather events. Australas Psychiatry. 2025;33(2):220–7. 39752293 10.1177/10398562241312865 PMC11982582

[56] Albert S, Leon JX, Grinham AR, Church JA, Gibbes BR, Woodroffe CD. Interactions between sea-level rise and wave exposure on reef island dynamics in the Solomon Islands. Environ Res Lett. 2016;11(5):054011.

[57] Gibson KE, Barnett J, Haslam N, Kaplan I. The mental health impacts of climate change: findings from a Pacific Island atoll nation. J Anxiety Disord. 2020;73:102237. 32485590 10.1016/j.janxdis.2020.102237

[58] Mathieu S, Leo D, Koo YW, Leske S, Goodfellow B, Kõlves K. Suicide and suicide attempts in the Pacific Islands: a systematic literature review. Lancet Reg Health. 2021;17:100246. 10.1016/j.lanwpc.2021.100283 PMC8495100 34734201

[59] WHO UNICEF Joint Monitoring Programme. Vanuatu 2023 multiple indicator cluster survey report. Geneva: WHO/UNICEF; 2024. https://washdata.org/reports/vanuatu-2023-mics-report. Accessed 2 Jan 2025.

[60] Burke M, Hsiang SM, Miguel E. Global non-linear effect of temperature on economic production. Nature. 2015;527(7577):235–9. 26503051 10.1038/nature15725

[61] Mach KJ, Kraan CM, Adger WN, Buhaug H, Burke M, Fearon JD, et al. Climate as a risk factor for armed conflict. Nature. 2019;571:193–7. 10.1038/s41586-019-1300-6. 31189956 10.1038/s41586-019-1300-6

[62] Choi HM, Heo S, Foo D, Song Y, Stewart R, Son J, et al. Temperature, crime, and violence: a systematic review and meta-analysis. Environ Health Perspect. 2024;132(10):106001. 10.1289/EHP14300. 39404825 10.1289/EHP14300 PMC11477092

[63] Ryan BJ, Franklin RC, Burkle FM Jr, Aitken P, Smith E, Watt K, et al. Identifying and describing the impact of cyclone, storm and flood related disasters on treatment management, care and exacerbations of non-communicable diseases and the implications for public health. PLoS Curr. 2015. 10.1371/currents.dis.62e9286d152de04799644dcca47d9288. 10.1371/currents.dis.62e9286d152de04799644dcca47d9288 PMC4593706 26468423

[64] Campbell JR. Development, global change and traditional food security in Pacific Island countries. Reg Environ Change. 2015;15(7):1313–24.

[65] Ferguson CE, Tuxson T, Mangubhai S, Jupiter S, Govan H, Bonito V, et al. Local practices and production confer resilience to rural Pacific food systems during the COVID-19 pandemic. Mar Policy. 2022;137:104954. 35035031 10.1016/j.marpol.2022.104954 PMC8746868

[66] Thow AM, Heywood P, Schultz J, Quested C, Jan S, Colagiuri S. Trade and the nutrition transition: strengthening policy for health in the Pacific. Ecol Food Nutr. 2011;50(1):18–42. 21888586 10.1080/03670244.2010.524104

[67] Troubat N, Sharp MK. Food consumption in Kiribati based on analysis of the 2019/20 household income and expenditure survey. Tarawa: FAO; 2021. 100. https://openknowledge.fao.org/items/c261119e-5445-41d9-8939-d7343a971327.

[68] Charlton K, Russell J, Gorman E, Hanich Q, Delisle A, Campbell B, et al. Fish, food security and health in Pacific Island countries and territories: a systematic literature review. BMC Public Health. 2016;16:285. 27009072 10.1186/s12889-016-2953-9 PMC4806432

[69] Radway K, Manley M, Mangubhai S, Lalavanua S, Caginitoba A, Dulunaqio S, et al. Impact of tropical cyclone winston on community fisheries in Fiji. Suva: Wildlife Conservation Society; 2016.

[70] Bell JD, Kronen M, Vunisea A, Nash WJ, Keeble G, Demmke A, et al. Planning the use of fish for food security in the Pacific. Mar Policy. 2009;33(1):64–76.

[71] Magel JMT, Burns JHR, Gates RD, Baum JK. Effects of bleaching-associated mass coral mortality on reef structural complexity across a gradient of local disturbance. Sci Rep. 2019;9(1):2512. 30792432 10.1038/s41598-018-37713-1 PMC6385266

[72] Baker AC, Glynn PW, Riegl B. Climate change and coral reef bleaching: an ecological assessment of long-term impacts, recovery trends and future outlook. Estuar Coast Shelf Sci. 2008;80(4):435–71.

[73] Lam VWY, Allison EH, Bell JD, Blythe J, Cheung WWL, Frölicher TL, et al. Climate change, tropical fisheries and prospects for sustainable development. Nat Rev Earth Environ. 2020;1(9):440–54.

[74] Hoegh-Guldberg O, Mumby PJ, Hooten AJ, Steneck RS, Greenfield P, Gomez E, et al. Coral reefs under rapid climate change and ocean acidification. Science. 2007;318(5857):1737–42. 18079392 10.1126/science.1152509

[75] Bell JD, Ganachaud A, Gehrke PC, Griffiths SP, Hobday AJ, Hoegh-Guldberg O, et al. Mixed responses of tropical Pacific fisheries and aquaculture to climate change. Nat Clim Chang. 2013;3(6):591–9.

[76] Chinain M, Gatti CMi, Martin-Yken H, Roué M, Darius HT. Ciguatera poisoning: an increasing burden for Pacific island communities in light of climate change. In: Botana LM, Louzao MC, Vilarino N, editors. Climate Change and marine and freshwater Toxins. Berlin: De Gruyter; 2020. p. 369–428.

[77] Lafferty KD, Mordecai EA. The rise and fall of infectious disease in a warmer world. F1000Res. 2016;5:2040. 10.12688/f1000research.8766.1 PMC4995683 27610227

[78] Georgeou N, Hawksley C, Wali N, Lountain S, Rowe E, West C, et al. Food security and small holder farming in Pacific Island countries and territories: a scoping review. PLoS Sustain Transform. 2022;1:e0000009.

[79] Mueller W, Zamrsky D, Essink GO, Fleming LE, Deshpande A, Makris KC, et al. Saltwater intrusion and human health risks for coastal populations under 2050 climate scenarios. Sci Rep. 2024;14(1):15881. 38987576 10.1038/s41598-024-66956-4 PMC11237024

[80] Tui S, Fakhruddin B. Food for thought: Climate change risk and food (in)security in Tuvalu. Prog Disaster Sci. 2022;16:100255.

[81] Barnett J. Dangerous climate change in the Pacific Islands: food production and food security. Reg Environ Change. 2011;11(S1):229–37.

[82] Lauer M, Albert S, Aswani S, Halpern BS, Campanella L, La Rose D. Globalization, Pacific Islands, and the paradox of resilience. Glob Environ Change. 2013;23(1):40–50.

[83] Dacks R, Ticktin T, Jupiter SD, Friedlander AM. Investigating the role of fish and fishing in sharing networks to build resilience in coral reef social-ecological systems. Coast Manage. 2020;48(3):165–87.

[84] Taylor S. The vulnerability of health infrastructure to the impacts of climate change and sea level rise in small island countries in the South Pacific. Health Serv Insights. 2021;14:11786329211020856. 34158800 10.1177/11786329211020857 PMC8186115

[85] Solomon Islands National Statistics Office. Solomon islands 2012/13 household income and expenditure survey national analytical report. Honiara: Solomon Islands National Statistics Office; 2015. Report No.: 1.

[86] Hammad K, Faavae LT, Taufilo A, Leong M, Nasila V. Responding to COVID-19 on the outer islands of Tuvalu. West Pac Surveill Response J WPSAR. 2024;15(2):1–5. 38919385 10.5365/wpsar.2024.15.2.1080 PMC11194254

[87] WHO Regional Office for the Western Pacific. Protecting health in Small Island Developing States: the state of the evidence on climate change and health. Manila: WHO Regional Office for the Western Pacific; 2021. https://iris.who.int/handle/10665/349700.

[88] Pacific Islands Forum. The Pacific Security Outlook Report 2022–2023. Suva: Pacific Islands Forum Secretariat; 2022. https://forumsec.org/sites/default/files/2023-12/Pacific-Security-Outlook-Report-2022-2023.pdf.

[89] Government of Kiribati, Ministry of Finance and Economic Development. 2018 Fishing License Revenue. Tarawa: Government of Kiribati; 2018. https://mfed.gov.ki/node/88. Accessed 30 Jun 2025.

[90] Bell JD, Senina I, Adams T, Aumont O, Calmettes B, Clark S, et al. Pathways to sustaining tuna-dependent Pacific Island economies during climate change. Nat Sustain. 2021;4(10):900–10.

[91] WHO Regional Office for the Western Pacific. Pacific islands action plan on climate change and health. Manila: WHO Regional Office for the Western Pacific; 2018. https://iris.who.int/handle/10665/275484. Accessed 2 Jan 2025.

[92] Secretariat of the Pacific Regional Environment Programme. Pacific Adaptation to Climate Change (PACC) programme. Apia: SPREP; 2024. https://www.sprep.org/pacc. Accessed 30 Jun 2025.

[93] World Bank. Pacific Islands—Pacific Resilience Program Project. Washington, DC: World Bank; 2017. https://documents.worldbank.org/en/publication/documents-reports/documentdetail/en/231881468001190037. Accessed 30 Jun 2025.

[94] Benjamin L, Thomas A. 1.5 to stay alive? AOSIS and the long term temperature goal in the paris agreement. Rochester: Social Science Research Network; 2016. https://papers.ssrn.com/abstract/3392503. Accessed 30 Jun 2025.

[95] International Institute for Sustainable Development. Anticipating ICJ advisory opinion on climate change. SDG Knowledge Hub; 2023. https://sdg.iisd.org/commentary/policy-briefs/anticipating-icj-advisory-opinion-on-climate-change/. Accessed 30 Jun 2025.

[96] Pacific Community Statistics for Development Division. Member countries. Noumea: Pacific Community; 2024. https://sdd.spc.int/all-countries. Accessed 30 Jun 2025.

